# Resilience, Stress, Well-Being, and Sleep Quality in Multiple Sclerosis

**DOI:** 10.3390/jcm12020716

**Published:** 2023-01-16

**Authors:** Anne Marie Novak, Shahar Lev-Ari

**Affiliations:** Department of Health Promotion, School of Public Health, Sackler Faculty of Medicine, Tel Aviv University, Tel Aviv 6997801, Israel

**Keywords:** multiple sclerosis, resilience, stress, sleep, fatigue, psychological well-being, satisfaction with life, happiness, MS, Israel

## Abstract

(1) Background: Multiple Sclerosis (MS) is a chronic, progressive, immune-mediated disorder that affects the Central Nervous System and is the most common cause of non-traumatic neurological disability in young adults. The study aimed to assess the levels of stress, resilience, well-being, sleep quality, and fatigue in Israeli people with MS (PwMS), and to examine the associations between these factors and the sociodemographic and clinical characteristics. These factors had never before been studied in conjunction in PwMS, nor had they been systematically addressed in Israel, the unique geopolitical situation of which may pose unique challenges. (2) Methods: This was a survey-based, cross-sectional study conducted through an Internet platform. (3) Results: Israeli PwMS who participated in the study were experiencing relatively high levels of stress and low resilience, poor sleep quality, and severe fatigue. The analysis revealed significant associations between resilience and stress, well-being, and anxiety, as well as stress and well-being, resilience, sleep quality, fatigue, and Clinically Isolated Syndrome (CIS). (4) Conclusions: the Israeli PwMS who participated in the study were experiencing higher levels of stress, lower resilience and worse sleep quality than PwMS in other countries, as compared to results previously reported in literature. The findings of this study ought to serve as a call to action for the MS care providers in Israel and warrant further research into the possible causes of the phenomenon and strategies to address it.

## 1. Introduction

Multiple sclerosis (MS) is a chronic, progressive, immune-mediated disorder that affects the central nervous system and the most common cause of non-traumatic neurological disability in young adults [1]. The progression of MS is not linear, and it is not yet clear what causes a benign course of disease in some patients and a rapid, debilitating course in others [1]. In addition to physical impairment and pain, people with MS (PwMS) often experience increased fatigue, cognitive difficulties, poor sleep quality, chronic stress, mood disturbances, lowered quality of life and resilience, and reduced well-being [2,3,4,5]. Large studies identified a correlation between perceived stress and depressive symptoms in PwMS. Chronic stress, as well as acute stressful events, were also recognized as potential triggers for MS exacerbations [6,7].

As of today, many MS patients receive innovative care focused on preventing exacerbations and slowing the disease progression [8,9]. However, the treatment they receive is centered around the physical aspects of the disease, while a more integrative and positive model of care might be of substantial benefit to the patients, most of whom are diagnosed in early adulthood and suffer from daily symptoms of the disease [3]. Disease-modifying therapies (DMTs) slow the disease progression, but they do not cure it, and they are not intended to aid PwMS with the associated psychological and emotional challenges. Yet, little attention is devoted to the psychological health, well-being, stress levels, sleep quality, and resilience of PwMS, all of which may greatly affect their day-to-day life. Therefore, we are obliged to inquire into the prevalence of these issues, as a possible foundation for creating a better, all-encompassing standard of care—for the patients to feel empowered, optimistic, and resilient when facing the challenge of building a life with a chronic, unpredictable disease without a cure.

Stress, defined as “a state in which homeostasis is actually threatened or perceived to be so” [10], has long been recognized as one of the major factors at play in MS etiology and progression [7]. In the experience of many PwMS, their disease onset or exacerbation immediately followed stressful events such as the death of a loved one, pressure at work, financial strain, relationship difficulties, or university exams [11,12]. However, stress described as common but constant might be more likely to contribute to the disease [13], and it appears that PwMS are more stressed than the general population [5]. Resilience—that is, the ability to withstand crises, adapt when faced with stressors and adversity, and recover quickly—might be one of the most important characteristics of PwMS who adapt and thrive despite this chronic, unpredictable, and potentially debilitating illness. Nonetheless, PwMS were shown to have a lower resilience score than the general population and primary care patients in large studies conducted in Canada and Australia [14,15]. Since developing and maintaining resilience can be of great import when facing chronic illness, impacting both everyday life and health outcomes [16], there is a theoretical reason to believe that resilience might be more strongly correlated with well-being in PwMS that in the general population.

A growing body of evidence points to the link between high levels of well-being and health outcomes, including reducing the risk of disease and physical and mental health improvements both in healthy subjects and the chronically ill [17,18,19]. In the general population, there exists a strong negative correlation between stress levels and psychological well-being [20,21], yet the association has not yet been established in PwMS, despite the role of stress in MS etiology and progression [7]. Developing and maintaining high levels of well-being in PwMS could contribute to their general health and lower their risk of depression [19].

Poor sleep quality is one of the major modifiable risk factors for cognitive decline [22,23]; in PwMS it has been specifically linked to diminished visual and verbal memory, executive function attention, and processing speed [24,25]. PwMS are very likely to suffer from sleep disturbances [26,27]: studies report that approximately half of PwMS may be suffering from poor sleep quality [28,29]. Despite this, sleep abnormalities remain underdiagnosed in PwMS [30] and sleep assessment is not used in most MS care centers [27]. Further, poor sleep quality is associated with fatigue, the most common symptom of MS, experienced by at least 75% and up to 90% of PwMS, and often perceived as more debilitating than pain and physical disability [31,32]. Fatigue in MS is also associated with higher rates of depression, anxiety, and reduced well-being [2,33].

The study aimed to assess the levels of stress, resilience, well-being, sleep quality, and fatigue in Israeli PwMS, and to examine the associations between these factors and the sociodemographic and clinical characteristics. While the variables studied here have been examined in the past in PwMS [5,14,28,33], the body of research remains relatively small and scattered, and they have never before been examined in conjunction. A higher rate of MS exacerbations was reported in Israel during the Israeli-Lebanese war, especially in PwMS who reported higher stress levels [34], pointing to the possible unique effect of the Israeli geopolitical environment on one’s experience of MS. However, to the best of our knowledge, the subjective well-being of Israeli PwMS was directly measured only once, in 2010, in the context of a specific DMT [35]. The study offers up-to-date, reliable data, including on the associations between the factors studied.

## 2. Materials and Methods

### 2.1. Study Design

This was a survey-based, cross-sectional study conducted through internet platforms for PwMS.

### 2.2. Study Setting and Study Population

The participants were invited through the private Facebook and WhatsApp groups managed by the Israeli MS Fund and MS Israel, two non-profit organizations that support Israeli PwMS and their families. Participation was voluntary and not rewarded in any form, and the participants were informed of the nature and topic of the study both in the invitation to participate and on the landing page for the survey. The survey was anonymous and no identification data was collected. The participants were free to abandon the online questionnaire at any point and could leave any of the questions unanswered. The study was conducted under a research protocol approved by the Tel Aviv University Ethics Committee, granted on 4 November 2021, no. 0004023-1. The recruitment began on 1 August 2022, and ended on 31 August 2022, upon reaching the required sample size. The survey was published and made available online in its entirety through Qualtrics, a highly secure cloud-based platform for creating and distributing web-based surveys based in the United States and licensed by Tel Aviv University. The inclusion criteria were as follows: (a) age above 18; (b) residence in Israel; (c) the ability to answer independently in Hebrew; (d) a confirmed diagnosis of multiple sclerosis, following the 2017 update to the McDonald criteria, and including the clinically isolated syndrome (CIS) [36]. The inclusion criteria, including the confirmed diagnosis of MS, were self-assessed at the beginning of the survey and a negative answer led to an automatic termination of the survey.

### 2.3. Study Tools and Outcome Measures

The dependent variables of the study included levels of perceived stress, resilience, psychological and subjective well-being (satisfaction with life), sleep quality, and fatigue, quantified through validated questionnaires. The explanatory variables consisted of sociodemographic and clinical factors. The short socioeconomic questionnaire was composed of six questions regarding one’s age, gender, marital status, family income, education level, and current occupation (including disability and leave of absence due to illness, following previous studies on PwMS [15]). The self-reported clinical factors examined were: MS clinical disease course (RRMS, PPMS, SPMS, CIS, unknown); description-based, self-assessed daily-functioning and disability level (based on descriptions previously used in studies on PwMS [37], and translated into Hebrew); disease-modifying treatment status; time since diagnosis; time since the last exacerbation; having experienced MS symptoms in the past week; the presence of comorbidities and mental health conditions (the survey included depression, anxiety, and a free text space for other diagnoses); receipt of psychological counseling following the MS diagnosis; and participation in MS support groups. The questions regarding the time since the last exacerbation and the MS symptoms experienced were followed by their accepted definitions and examples, with an exacerbation defined as an occurrence of a new symptom(s) or a worsening of a previously existing symptom(s) of MS that lasts at least 48 h. The survey questions are available in their entirety in the Supplementary File. The current study included a self-reported CIS diagnosis as a sub-type of MS in addition to RRMS, PPMS and SPMS. While the agreement levels between CIS as diagnosed by a physician and self-reported by a person with MS have not been tested, other studies have shown good agreement levels between MS type as defined by a physician and the self-reported MS type (Kappa = 0.62) [38]. The participants were additionally asked if they were recommended psychological counseling by their neurologists.

The 10-item version of the Perceived Stress Scale (PSS-10) was employed for the measurement of perceived stress levels. The questionnaire was developed by S. Cohen et al. in 1983 and has been in wide use since for the assessment of perceived stress levels [39]. The scale quantifies the stress perceived by respondents in the last month, with each of the 10 questions addressing how often they felt a certain way (e.g., “In the last month, how often have you felt nervous or stressed?”), on a scale from 0 (never) to 4 (very often). It has been validated in various populations, and it has good internal consistency and test-retest reliability, as well as concurrent, convergent, and construct validity [40]. It is widely used for the assessment of stress levels in PwMS [5]. The Hebrew translation was developed by Oren Lahak at the Meir General Hospital in Kfar Saba, Israel [41].

The 10-item short form Connor Davidson Resilience Scale (CD-RISC) was used to measure resilience. This scale measures the degree to which respondents can adapt to challenges (e.g., “I am able to handle unpleasant or painful feelings like sadness, fear, and anger”). The scale is the most common and internationally-validated method for measuring resilience, including in PwMS [15,42,43]. It was developed and published in 2003 by K. Connor and J. Davidson [44], and its briefer, 10-item version was validated in 2009, revealing almost identical mean scores in the general US population to the full 25-item version [45]. The scale was shown to be internally consistent, with good test-retest reliability and validity across various languages and cultures [46,47,48]. The Hebrew translation has been employed in various studies conducted in Israel [49].

The 18-item short form of Ryff’s Psychological Well-being Scale (PWB) was employed for the measurement of six dimensions of psychological well-being as defined by C. Ryff: autonomy, environmental mastery, personal growth, positive relationships with others, purpose in life, and self-acceptance [18,50]. The short version of the questionnaire consists of 18 statements with which the respondents are asked to agree or disagree on a 7-point scale (e.g., “In many ways I feel disappointed about my achievements in life”). It has been widely used since its conception and validated in different countries and groups [51,52], including PwMS [4]. Subjective well-being was assessed using the Satisfaction with Life Scale (SWLS), a validated, 5-item questionnaire, employing the same 7-point scale, that is most widely used for the measurement of subjective well-being, including in PwMS [53,54]. Developed by Ed Diener in 1985, the scale has high internal consistency and good test-retest correlation [53,55] and has been validated in Hebrew in the Israeli population [56]. In PwMS, the scale had revealed diminished subjective well-being [54].

The Pittsburgh Sleep Quality Index (PSQI), an internationally-used, validated, and reliable questionnaire for self-reporting of sleep quality over the period of one month, was used for the measurement of sleep quality in the study sample. It was shown to have adequate and good test-retest reliability and internal consistency and was validated in different groups and a large variety of languages, including Hebrew [57,58]. Created in 1988, it consists of 19 items that assess sleep quality, including its duration, efficiency, and disturbance, judged by the respondent on a 0–3 scale [59]. Scores above 5 are considered an indication of poor sleep quality. Fatigue severity was assessed using Krupp’s Fatigue Severity Scale (FSS), a 9-item questionnaire, with each statement measured on a 7-point Likert scale, for the identification of fatigue and measurement of its impact. The questionnaire was developed specifically for PwMS and lupus patients in 1989 and is widely used to this day in this population, as well as in chronic fatigue syndrome patients [60,61]. The scale was validated and shown to have very high reliability and acceptable internal consistency [31]. Its results correlate with other questionnaires used for the assessment of fatigue [31]. The results can be presented either as a sum (with a range of 1 to 63) or a mean of scores (with a range of 1 to 7), with higher scores indicating greater fatigue. It was translated into Hebrew for the purpose of this study and retranslated into English for accuracy verification by a colleague.

### 2.4. Sample Size

The sample size calculation was based on the reported prevalence of high levels of stress in PwMS. A 2021 study found that 47.7% of PwMS experienced high levels of stress (5), measured and quantified using the 10-item Perceived Stress Scale (PSS-10), the questionnaire to be used in this study. A sample size calculation conducted using WinPepi software, version 11.65, revealed a required sample size of 150 respondents, using an assumed rate of high stress at 48 per 100, at a 95% confidence level, with an accepted difference of 8 per 100. To account for missing data and outliers, the target sample size was increased by 20% to 201 respondents to yield a confidence interval width of up to 16 per 100, if the observed rate is between 30 and 70.

### 2.5. Statistical Analysis

The collected data was transferred into IBM SPSS software, version 28. Descriptive statistics were used to assess the characteristics of the study sample. The distribution of the variables was assessed using the Kolmogorov-Smirnov tests. Independent *t*-tests and ANOVA were used to assess the associations between the dependent and binary explanatory variables of the study that had a normal distribution. Mann-Whitney and Kruskal-Wallis tests were utilized for the variables that did not follow a normal distribution. To account for the number of *t*-tests performed for each of the dependent variables, the required *p*-value for statistical significance was reduced to *p* = 0.008, through the Bonferroni correction. Pearson’s and Spearman’s correlation coefficients were calculated for each of the possible pairs of dependent variables as well as age. Stepwise multivariate regression models were employed to determine the associations between the factors measured. The gender and age of the participants were included in the final regression models due to their role in the MS course and progression. The multicollinearity of the variables was examined in each model.

The sample size calculation was based on the reported prevalence of high levels of stress in PwMS.

## 3. Results

### 3.1. Participants Characteristics

In total, 270 people responded to the invitation to participate in the survey, of whom 259 met the inclusion criteria: age above 18, residence in Israel, the ability to answer independently in Hebrew, and a confirmed diagnosis of multiple sclerosis. Not all participants completed the whole questionnaire, but each of the separate scales received at least 196 complete and valid responses (223 valid responses for the PSS-10, 221 for CD-RISC, 202 for SWLS and for PWB, and 196 for PSQI and FSS). The majority of participants were women (79.6%), as was expected, given that the prevalence of MS is much higher in women, at a ratio of 3:1 [62]. The average age was 41.7 with a range of 20 to 73, and most respondents were either married or in serious relationships. The income distribution followed the normal curve. The majority of respondents (54.4%) held an academic degree, a proportion close to the national average of 46%, with Israeli women being more likely to have attained tertiary education, according to the most recent OECD report [63]. Most participants reported working full-time (34.8%) but a significant proportion was fully disabled and unemployed (23.6%). The complete distribution of background factors is presented in Table 1.

Most participants were diagnosed with the relapsing remitting form of MS (63.1%), followed by primary progressive MS (20.1%), secondary progressive MS (9.4%), and clinically isolated syndrome (5.7%); 11.5% of people did not know which form of MS they were diagnosed with. The amount of time since one’s MS diagnosis and the last exacerbation varied. The majority of respondents reported receiving disease-modifying therapy (83.2%). Most of the people queried (86.1%) experienced MS symptoms during the previous week and were diagnosed with at least one comorbidity (54%), the most common of which were hypercholesterolemia, a different autoimmune illness, and thyroid disorders. Additionally, 16.4% of participants were diagnosed with depression and 16.0% with anxiety. The majority suffered from some level of disability, self-assessed using a scale presented in detail in Table 2. The majority of participants did not see a therapist following their diagnosis (53.7%), did not participate in group support sessions (77.9%), and were not recommended to see a therapist by their neurologists (58.6%). The complete distribution of factors related to MS and general health in the study population is presented in Table 3.

### 3.2. Relationships between the Psychosocial Factors, Sleep Quality and Fatigue and the Sociodemographic and Clinical Characteristics

The mean score for perceived stress was 19.6 (SD = 6.4), classified as “moderate stress” by the authors of the scale [39]. The mean score for resilience stood at 22.1 (SD = 7.8) out of a possible 40 points, and for well-being at 88.3 (SD = 13.8), out of 126 points possible. Of the six dimensions of psychological well-being, environmental mastery had the lowest score of 13.6 (SD = 3.9), followed by purpose in life (14.3, SD = 3.0), positive relationships with others (14.4, SD = 3.6), self-acceptance (14.6, SD = 4.0), autonomy (15.2, SD = 3.2), and personal growth (16.2, SD = 2.7). The mean satisfaction with life was 20.8 (SD = 7.3), or “neutral” [53]. The average sleep quality score was 8.9 (SD = 4.0), indicating poor sleep quality [59]; the average score for fatigue was 48.9 (SD = 11.5), indicating severe fatigue [60]. Some 86.1% of the study participants had poor sleep quality, and 71.4% were experiencing severe fatigue, defined as a score above 45 on the FSS [64]. The Cronbach’s Alpha values ranged between 0.823 and 0.924 for all scales except the Pittsburgh Sleep Quality Index, with a score of 0.659. The exact values are presented in the Supplementary File.

PwMS who were fully employed had the most favorable results across all factors, with the opposite results among the unemployed or fully disabled and unemployed. The effect sizes indicated that the magnitude of the correlation was largest between employment and fatigue, sleep quality and satisfaction with life (ω^2^ > 0.065). People reporting a below-average family income had the least favorable results for each of the factors examined (*p* < 0.001, ω^2^ > 0.069; *p* = 0.007 and ω^2^ = 0.060 for sleep quality). An examination of the effect sizes revealed that holding an academic degree did not have a medium or large effect on one’s resilience, well-being, and satisfaction with life, despite the significant *p*-values. PwMS who were married or in serious relationships had greater satisfaction with life (*p* = 0.004, ω^2^ = 0.066). Time since diagnosis did not have a large effect on the factors examined. Those who most recently had an MS exacerbation had greater fatigue (*p* = 0.002, ω^2^ = 0.069). Experiencing MS symptoms during the previous week was associated with higher stress levels (*p* = 0.006, d = −0.560) and fatigue severity (*p* < 0.001, d = −1.102), and lower satisfaction with life (*p* = 0.005, d = 0.540) and sleep quality (*p* < 0.001, d = −0.821).

Having at least one comorbidity was associated with greater stress levels (*p* = 0.003) and fatigue (*p* = 0.005), although the effect sizes were low to medium (d = −0.402 and d = −0.371 respectively). The self-rated disability levels, divided into six categories as presented in detail in Table 2, were significantly associated with fatigue (*p* < 0.001, ω^2^ = 0.125) and sleep quality (*p* < 0.001, ω^2^ = 0.107) of the participants. A third of the participants were diagnosed with a mental health condition. Clinical depression, present in 16.4% of the study population, and clinical anxiety, present in 16% of the study population, were significantly associated with higher stress levels and fatigue (*p* < 0.001), as well as lower resilience (*p* < 0.001), well-being (*p* < 0.001), satisfaction with life (*p* < 0.001), and sleep quality (*p* = 0.007 and *p* = 0.034, respectively), as compared to individuals without these diagnoses. An analysis of the effect sizes revealed a medium to large magnitude of the effect of both anxiety and depression on stress, resilience, well-being, fatigue, and satisfaction with life (0.631 < d > 1.0). Having seen a therapist following the MS diagnosis was associated with higher levels of stress and fatigue and lower resilience, although the effect reached the medium threshold only for fatigue (d = −0.511).

Gender, types of MS, being on a DMT, and having participated in support groups were not significantly associated with any of the dependent variables studied. People who were not able to say which type of MS they had appeared to have slightly higher perceived stress levels and fatigue severity, as well as lower resilience and well-being than other categories. Not knowing one’s type of MS was not significantly associated with their education or income.

### 3.3. Correlations between the Participant’s Stress, Resilience, Well-Being, and Sleep Quality

Table 4 summarizes the correlations between the dependent variables of the study as well as age. The highest observed correlation was between satisfaction with life and well-being (0.775, *p* < 0.001), two related concepts. Moderately high positive correlations were also observed between well-being and resilience (0.699, *p* < 0.001), fatigue and perceived stress (0.543, *p* < 0.001), and satisfaction with life and resilience (0.573, *p* < 0.001). Moderately high negative correlations were seen between stress and resilience (−0.641, *p* < 0.001), well-being (−0.627, *p* < 0.001), and satisfaction with life (−0.559, *p* < 0.001). Moderate negative correlations were measured between fatigue and resilience (−0.463, *p* < 0.001), well-being (−0.418, *p* < 0.001), and satisfaction with life (−0.403, *p* < 0.001).

### 3.4. Relationship between Perceived Participant’s Stress, Resilience, Well-Being, and Sleep Quality: A Multivariable Linear Model

In a multivariable linear model presented in Table 5, perceived stress was associated with resilience, sleep quality, fatigue, and a CIS diagnosis, as well as two subscales of psychological well-being: environmental mastery and self-acceptance. Greater resilience, environmental mastery, and self-acceptance were associated with lower stress levels. The final model explained 66% of the variance in stress (R^2^ = 0.655). None of the variables demonstrated sufficient collinearity to be removed.

In an additional multivariable linear model for resilience, presented in Table 6, resilience was associated with perceived stress and three psychological well-being subscales, namely autonomy, environmental mastery, and personal growth. The final model, which explains 61% of the variance in resilience (R^2^ = 0.607), showed a negative association between stress and diagnosed anxiety with resilience and a positive one between the dimensions of well-being and resilience.

The third multivariable linear model for sleep quality, presented in Table 7, explained 33% of the variance in variable (R^2^ = 0.329). The third model included perceived stress, experiencing symptoms of MS in the previous week, having had an MS exacerbation in the previous three months, a below-average income, and one aspect of psychological well-being: autonomy. Additionally, having no perceived disability was associated with better sleep quality. Due to their relevance in MS, age and gender are also reported in the final models despite not being statistically significant. The complete statistical analyses for all three regression models are available in the Supplementary File.

## 4. Discussion

This cross-sectional study was, to the best of our knowledge, the only study to date that examined perceived stress, well-being, resilience, fatigue, and sleep quality in conjunction, in a large sample of PwMS. These five factors are interconnected and conjointly create a more complete picture of one’s everyday functioning with MS, beyond the physical symptoms. Further, they had never been systematically addressed in Israel, the geopolitical situation of which may pose unique challenges. The results reveal relatively low levels of resilience and high levels of stress, and a very high prevalence of poor sleep quality and severe fatigue among Israeli PwMS, as compared to the healthy population and PwMS in other countries. Resilience was significantly associated with stress, well-being, and anxiety, while stress was correlated with lower well-being, resilience and sleep quality, greater fatigue, and CIS. These results highlight the interplay between the factors studied and point to possible strategies to address the concern.

### 4.1. Resilience

The mean level of resilience, quantified using the CD-RISC-10 scale, was 22.1 (SD = 7.8), out of 40 points possible. To the best of our knowledge, this is the lowest score for resilience in PwMS observed to date globally, and the first study to measure the resilience of PwMS in Israel. So far, the lowest resilience in PwMS, a score of 23.0 (an approximation, after conversion to the CD-RISC-10 Scale from the CD-RISC-25), was reported in Iran [65]. In a recent study conducted in Canada, the average resilience score in PwMS was 72.4, approximately 28.9 on the CD-RISC-10 [15], and in Australia, the reported score was 27.0 [14]. Resilience scores were found to differ between countries [14,15], as it is influenced by one’s life circumstances [44]. Importantly, in all the studies mentioned, PwMS scored lower than the general population in each country [14,15,65]. As K. Turpin hypothesized following these findings in Canada, the lowered resilience in PwMS may be caused by stressors that are impossible to control or change [15]: MS remains an unpredictable disease, and coping with unpredictability lowers resilience, as has been shown in other populations [66].

In the current study, resilience was associated with stress, anxiety, and three aspects of psychological well-being: autonomy, environmental mastery, and personal growth. The model was adjusted for age and gender and explained 61% of the variance in resilience. These same three subscales of well-being, as defined by C. Ryff [50], were identified as predictors of greater resilience in the study on resilience in Canadian PwMS [15]. Autonomy and environmental mastery appear of special import in PwMS as they include one’s ability to make autonomous choices and remain independent while managing one’s environment, all despite the challenging, absorbing, and potentially debilitating nature of MS. Several trials conducted to date show that resilience is not stagnant, and it can be improved through interventions [67,68]. Both physical and cognitive rehabilitation programs improve and maintain the independence and autonomy of PwMS [69,70], and psychological support may further strengthen their perception of environmental mastery and the ability to grow and thrive [71], thus improving resilience. One’s perception of environmental mastery may be negatively affected by the struggle to access proper medical and rehabilitative care as well as disability benefits [72], both of which remain especially challenging in Israel [73,74,75], and improvements in these areas may further lead to increased resilience in Israeli PwMS. Anxiety was found to be associated with lower resilience, as could be expected, given that otherwise healthy people with generalized anxiety disorder were also found to have lower resilience [44,46]. This study confirms the recently-reported links between high resilience levels in PwMS and a lower risk for anxiety, lower stress levels, and greater well-being, with other studies reporting additional benefits in motor strength and endurance [68,76,77]. Because of the impact of resilience on psychological and physical health, and because it can be improved [78], resilience deserves greater attention in MS care. Support groups have proven especially effective at resilience-building [78,79,80,81], and they remain some of the most accessible and cost-efficient tools for mental health improvement [82].

### 4.2. Stress

In this study, the mean level of perceived stress was 19.6 (SD = 6.4), a result classified by the authors of the Perceived Stress Scale as “moderate stress” [39]. This result was slightly higher than recently observed in PwMS in Poland (18.88 [5]), and much higher than was reported earlier in the United States (16.55 [37]). PwMS in the current study were considerably more stressed than the healthy population, in which the reported stress levels were 13.02 [83] and 15.06 [84], or 15.32 at the height of the COVID pandemic in Israel [85]. As previous studies have reported and this study confirms, PwMS appear to experience significantly higher levels of stress than the general population [83]. In the case of Israeli PwMS, stress might be exacerbated by extreme circumstances, including an ongoing war and rocket fire: during the 33 days of the 2006 war between Hezbollah and Israel, the Carmel Medical Center in Haifa observed significantly more exacerbations in its patients than usual, and the patients who suffered exacerbations were more likely to have reported intense stress [34]. During the data collection for this study, Israel was yet again experiencing unrest, with over 400 rockets fired on Israel over a single weekend in August 2022 [86]. Israeli PwMS appeared to be more stressed than the healthy Israelis whose stress levels were measured at the beginning of the COVID-19 pandemic [85]. A similar finding was reported from Italy, where, during the pandemic, the stress levels of PwMS increased more than in the healthy respondents [87], revealing the vulnerability of this population.

In the current study, as revealed by a linear regression model that explained 66% of the variance in the variable and was adjusted for age and gender, perceived stress was related to poor sleep quality and fatigue, while greater resilience, environmental mastery, and self-acceptance (two elements of psychological well-being, as defined by C. Ryff) reduced it. PwMS diagnosed with CIS—the initial stage of MS, and a diagnosis that follows its first episode—appeared to be experiencing more stress than those diagnosed with other courses of the disease. This observed effect might have been caused by the challenge of adapting to a new diagnosis and the unknown that it brings, similarly to what has been observed in people shortly after a cancer diagnosis [88]. The observed association here between stress and sleep quality was recorded in various studies [89], and appears of utmost importance—poor sleep quality in MS may lead to greater impairment [24,25,90], and one’s stress levels remain a modifiable risk factor [91,92,93]. Fatigue, one of the most common and debilitating symptoms of MS [31,32], was also associated with stress, a finding that aligns with the previous studies on the subject [94]. As was shown in the healthy population, stress may be one of the causes of fatigue [95,96], and thus efforts aimed at reducing the prevalence of stress in PwMS may have a positive effect on daily life with MS [31,32]. Further, the analysis revealed that increased resilience and some aspects of psychological well-being (environmental mastery and self-acceptance) reduced the stress levels of PwMS. This was an expected finding given the relationship between these concepts: resilience is the successful adaptation to challenging life experiences [97], while chronically increased stress precludes well-being [98]. Environmental mastery, or the sense of control over one’s life and circumstances, can mitigate stress [99], and appears uniquely relevant in people who are chronically ill and disabled. Similarly, self-acceptance may be especially challenging for PwMS, yet appears to have a protective effect in relation to stress [100], and its development may further reduce the stress experienced by PwMS.

It should be noted that stress appears to play a role in the pathogenesis of MS by dysregulating the immune response, increasing one of the Th1 cytokines and in turn impairing the balance between them and Th2 cytokines [7]. Chronic stress may in time lead to glucocorticoid resistance in the immune cells in PwMS [6]. Further, increased stress, as measured using PSS-10 that was utilized in this study, had been linked to poorer health practices, including sleeping fewer hours [101], as well as elevated cortisol levels, markers of aging, and pro-inflammatory cytokines [102]. The current study underlines the need to address the stress levels experienced by PwMS, and PwMS in Israel in particular. Behavioral interventions, mindfulness training, and coping strategies aimed at lowering stress were found to be effective in reducing the stress levels of PwMS, and may also lead to fewer brain lesions as well as greater well-being in this population [91,92,93,103].

### 4.3. Well-Being

The six-factor model of psychological well-being was developed by Carol Ryff who sought to unify the existing philosophical and psychological theories of what contributes to one’s happiness, well-being, self-actualization, and satisfaction with life [18]. No official “cut-offs” for the results have been approved and the interpretation of the results relies on comparisons [18]. The results in Israeli PwMS, at 88.3 points (SD = 13.8) out of 126 points possible, are more favorable than was recently reported in PwMS in Canada but not considerably so [15], and much more favorable than was reported in Iran [4]. Since the levels of well-being vary significantly between different countries, even those located in one geographical region [104], the difference is not entirely surprising. Israel has been consistently recognized as one of the happiest countries in the world, and it has been speculated that Israel’s high happiness levels are largely caused by the closeness, trust, and familial nature of the Israeli society [105], factors especially beneficial to PwMS who may rely on others to a greater-than-usual extent. Strengthening this point, the “positive relationships with others” element of psychological well-being in the current study was at a considerably higher level than in Canadian PwMS (14.4 next to 11.1), where it ranked the lowest of all subscales [15]. However, happiness does not equal psychological well-being, and to make any conclusions regarding the well-being of Israeli PwMS, one would have to consider the levels observed in the healthy Israeli population, and these are not available at present. Environmental mastery, related to the feeling of “being in charge” of one’s environment and everyday affairs and being able to make use of opportunities, ranked the lowest of all subscales at 13.6 points. This result might signify the increased difficulty of achieving this goal when affected by MS and its symptoms.

Resilience, stress, and fatigue were all significantly correlated with both the psychological and subjective well-being of Israeli PwMS. Subjective well-being, quantified in this study using the Satisfaction with Life scale, is a less comprehensive model of well-being that focuses on life satisfaction. In the current study, the mean satisfaction with life, at 20.8 points, was within the average range reported in healthy populations, but it was lower than previously reported both in Israeli [35] and American PwMS [54]. In a study conducted in Israel in 2010 that measured the satisfaction with life of PwMS treated with a specific DMT, the reported score was 23.9 [35]. While that result did not significantly differ from that of the healthy volunteers measured at that same time (25 points), the currently-reported score might signify a slightly decreased satisfaction with the lives of Israeli PwMS at this point in time. In this study, people who experienced MS symptoms in the previous week had significantly lower levels of satisfaction with life, a result consistent with previous reports [54]. This finding underlines the importance of addressing the ongoing symptoms of the disease—such as fatigue, pain, muscle weakness, brain fog, or paresthesia—as a way in which the healthcare professionals responsible for MS care may contribute to their patients’ general well-being. However, unlike the previous reports [35,54], time since diagnosis and being on a DMT did not contribute to the individuals’ perceptions of their well-being.

In this study, both psychological and subjective well-being were significantly associated with one’s income, employment, education, and marital status, with the highest scores among people in relationships and married, fully employed, earning above-average incomes, and with the highest educational achievement. All of these findings align with what was previously reported in healthy populations [106,107,108]. The results point to the need for making educational institutions and gainful employment more accessible to the chronically ill and disabled people—an issue that Israel has been struggling with [73]—as it can lead to improvements in their well-being. These findings further underscore the greater societal issues at play in determining one’s well-being, including in people faced with a life-changing diagnosis. Well-being has been linked in extensive studies to greater health and longevity [104], and its further study in PwMS and other chronically ill populations is warranted. While it is strongly influenced by the environment, circumstances, and personalities, a multitude of studies show it can be improved [17,109].

### 4.4. Sleep Quality and Fatigue

In the current study, 86.1% of PwMS had poor sleep quality, as defined by a score above 5.0 on the PSQI. The mean level of sleep quality was 8.9 (SD = 4.0). To the best of our knowledge, this is the highest prevalence of poor sleep quality in PwMS observed to date. The most recent studies on the subject reported lower rates, with poor sleep quality observed in about half of the study participants [110,111] and insomnia in 66.45% [112]. Sleep is especially important in PwMS because of its role in brain regeneration and regulation of inflammatory processes [25]. Poor sleep quality is related to cognitive decline, memory and attention problems, and lower processing speeds, all common and disturbing symptoms of MS as it progresses [24,25,113]. In the current study, poor sleep quality was observed across all the subcategories of PwMS examined, in all types of MS, both in the recently diagnosed and in those who have been diagnosed for over 20 years, in those who receive DMT and in those who do not. Worse sleep quality was observed in those who most recently experienced an MS exacerbation (*p* < 0.001, ω^2^ = 0.069), although this finding might be related to the lingering effects of a course of steroids [114,115]. Further, sleep quality appeared progressively worse with each of the disability subcategories (*p* < 0.001, ω^2^ = 0.125), and poor sleep quality was associated with depression, perceived stress, autonomy, and below-average income. These findings reveal a very significant burden on PwMS that often remains overlooked in MS care—sleep abnormalities are underdiagnosed in PwMS [30] despite their significant effects on one’s quality of life [112], and sleep assessment is not used in most MS care centers [27]. The elevated levels of poor sleep quality among Israeli PwMS may be related to the increased levels of stress in the Israeli society, yet a recent study that included 195,000 Israelis revealed an overall prevalence of sleep disorders—but not poor sleep quality specifically—that is similar to other Western countries [116]. The current finding warrants further research into both the possible causes of the phenomenon and the strategies to address it.

The mean score for fatigue, quantified using the FSS, was 48.9 points (SD = 11.5), and 71.4% the participants had severe fatigue, indicated by a total score above 45 on the FSS. These results are only slightly lower than what was identified in people with chronic fatigue syndrome [61], and indicate a life-changing impact of fatigue on one’s daily life with MS. This finding is similar to what was previously reported in this population: fatigue is one of the most common symptoms of MS and severe fatigue was found in other studies in approximately 70–80% of PwMS [64,117]. In this study, severe fatigue was found across all subtypes of MS and times since diagnosis, without significant differences. In line with other studies on the subject [64], people with PPMS were experiencing the highest levels of fatigue but in the current study, the differences were not significant. Having any level of disability, anxiety, or depression significantly increased the participants’ fatigue levels. Previous studies on the subject reported similar findings [64,117]. Next to the expectedly high levels of fatigue prevalence in the study population, the analysis revealed significant correlations between fatigue and stress, resilience and well-being of PwMS. Experiencing daily fatigue may drastically reduce one’s resilience [118] and in turn impair one’s well-being and the ability to cope with stress. Stress is also known to increase fatigue, through its effect on the mental load [95,96]. Prescribing physical activity [119], relaxation and mindfulness practices [120], stress reduction [95,96], cognitive behavioral therapy [121], and energy-preservation techniques [122] may go a long way in reducing the impact of fatigue in PwMS.

### 4.5. Mental Health

A third of the study participants reported having been diagnosed with a mental health condition, with 16.4% being diagnosed with depression, and 16% with anxiety. An estimated 8.4% of the US population has had at least one major depressive episode [123], and an estimated 6.1% of American adults experience moderate to severe symptoms of generalized anxiety disorder [124], rates considerably smaller than what was observed in this survey. However, it has been established that PwMS are at a much higher risk for depression and anxiety, with the prevalence estimates at 31% for depression and 22% for anxiety [125]. This study confirms the increased rate of mental health issues among PwMS in Israel, but the finding is limited by the self-report strategy for data collection, no additional assessment of the symptoms of depression and anxiety, and the lack of follow-up questions detailing the time and type of diagnosis and symptoms. It is likely that the true rates of depression and anxiety were higher and went undetected in this study due to its nature. In the current study, PwMS suffering from anxiety and depression had significantly higher levels of stress (*p* < 0.001) and fatigue (*p* < 0.001), as well as lower resilience (*p* < 0.001), well-being (*p* < 0.001), and sleep quality (*p* = 0.034 and *p* = 0.007, respectively), as compared to the study participants who were not diagnosed with either of these two conditions. There exists a documented association between depression, stress, and sleep quality in PwMS [89,126] which may be related to the perceived cognitive problems [89]. However, most of the respondents reported not seeing a therapist following their diagnosis and not participating in MS support group sessions, which are offered by the Israeli MS fund and are facilitated by qualified social workers. Further, 58.6% of the participants reported not having been recommended therapy by their neurologists. While the stress levels, resilience, and well-being appeared to be worse in those who have seen a therapist post-diagnosis, this effect appears to be related to the fact that those most affected were more likely to seek help. All in all, given the increased risk for depression and anxiety disorders in PwMS, in conjunction with the findings of this study regarding the high levels of stress and lowered resilience in this population, the need for better mental health care in MS appears fundamental. A Salutogenic approach—creating and supporting health, resilience, and well-being—could substantially benefit the tertiary prevention offered in MS centers in Israel.

### 4.6. Limitations

The findings of this study are limited by the cross-sectional nature of the data that precludes causal interference. To understand the causal pathways at play, longitudinal studies are needed. Further, the use of an internet-based survey for self-reporting might have led to the collection of inaccurate data, including on the type of MS, disability level, comorbidities, and disease duration. However, any such inaccuracies would be non-systematic, and previous studies showed good agreement between self-reported disease type and disability level and physician-reported data in PwMS [38]. Additionally, shorter—but validated and reliable—versions of the questionnaires were chosen for this study, to ensure a greater chance of accurate completion of the whole survey, especially given the study population’s particular risk for increased fatigue, including cognitive fatigue. The participants were volunteers who may have been interested in the concepts examined in the study, possibly due to reduced well-being, increased perceived stress, or similar factors. To reduce the impact of volunteer bias, data were collected from over two hundred participants, and the anonymity of volunteers was ensured and stressed in the invitation. Due to the inclusion criteria, the findings may not generalize to young PwMS (below the age of 18) as well as people who are cognitively impaired and/or severely disabled and thus not able to answer independently. Nevertheless, this study presents meaningful data on the stress levels, resilience, well-being, sleep quality, and fatigue of Israeli PwMS as well as the associations between these factors and background characteristics.

## 5. Conclusions

This cross-sectional study offers up-to-date data on the perceived stress, well-being, resilience, fatigue, and sleep quality of Israeli PwMS, and further research can be developed on its foundations, most importantly on interventions aimed at improving the quality of life of PwMS. The study sample included 223 Israeli PwMS of various ages, backgrounds, and clinical courses of MS, offering an in-depth picture of the variety of experiences of living with MS. The study found that Israeli PwMS had lower resilience, worse sleep quality and higher levels of stress than PwMS in other countries. Stress was correlated with worse sleep quality and fatigue, as well as well-being and resilience—pointing to the vital importance of stress management in MS, which should perhaps be routinely addressed by healthcare providers. Improving one’s resilience, shown to be possible through a range of interventions, may lead to lowered stress levels and improved well-being. The 86.1% prevalence of poor sleep quality found in this study underscores the need for thorough sleep quality assessments in standard MS care and for the employment of sleep-improvement techniques and therapies in PwMS. While limited, these findings ought to serve as a call to action for the MS care providers in Israel and worldwide, and warrant further research into the possible causes of the phenomena and strategies to address it.

## Figures and Tables

**Table 1:** Background Characteristics.

Background Characteristics	N (%) ^A^ or Mean (SD)	Stress ^B^ Mean (SD) *p*-Value t/F d or ω^2^		Resilience ^B^ Mean (SD) *p*-Value t/F d or ω^2^		Well-Being ^B^ Mean (SD) *p*-Value t/F d or ω^2^		SwL ^B^ Mean (SD) *p*-Value t/F d or ω^2^		Sleep Quality ^B^ Mean (SD) *p*-Value t/F d or ω^2^		Fatigue ^B^ Mean (SD) *p*-Value t/F d or ω^2^	
**Age**	41.7 (12.5)												
**Gender**													
Men	51 (20.4%)	19.2 (5.4)		22.5 (8.0)		85.6 (13.4)		21.7 (7.6)		9.2 (3.7)		45.5 (13.3)	
Women	199 (79.6%)	19.7 (6.6)	0.664	22.0 (7.7)	0.656	89.0 (13.9)	0.075	20.5 (7.2)	0.360	8.9 (4.1)	0.665	49.7 (10.9)	**0.043**
t			−0.435		1.651		−1.442		0.918		0.413		−2.034
Cohen’s d			−0.074		0.271		−0.252		0.159		0.077		−0.364
**Marital status**													
Single	47 (18.8%)	20.2 (6.4)		19.7 (7.5)		82.6 (11.7)		17.8 (7.5)		8.3 (4.7)		48.6 (13.6)	
In a serious relationship	38 (15.2%)	19.3 (6.7)		25.0 (6.7)		90.1 (11.5)		21.3 (6.7)		8.5 (4.2)		48.8 (11.5)	
Married	139 (55.6%)	19.1 (6.2)		22.6 (7.7)		90.4 (13.5)		22.4 (6.9)		8.8 (3.9)		48.0 (11.0)	
Divorced	25 (10.0%)	21.3 (7.1)		19.3 (8.4)		84.3 (12.8)		17.2 (7.3)		10.8 (3.2)		54.0 (9.6)	
Widowed	1 (0.4%)	17 (-)	0.579	22.1 (-)	**0.014**	97.0 (-)	**0.019**	17.0 (-)	**0.004**	11.0 (-)	0.141	47.0 (-)	0.289
F			0.720		3.220		3.016		4.568		1.446		1.257
ω^2^			−0.005		0.039		0.038		0.066		0.010		0.005
**Family income strata**													
Significantly above average	2 (0.8%)	23 (17)		21.0 (14.1)		89.5 (12.0)		25.0 (1.4)		14.5 (0.7)		48.0 (9.9)	
Above average	69 (27.6%)	17.5 (5.2)		25.5 (5.7)		94.8 (12.0)		24.2 (6.2)		8.2 (3.8)		44.4 (11.2)	
Average	134 (53.6%)	19.3 (6.4)		21.8 (7.8)		86.9 (13.6)		20.2 (7.1)		8.5 (3.9)		49.8 (11.2)	
Below average	35 (14.0%)	23.1 (5.3)		18.5 (7.4)		83.1 (14.4)		17.4 (6.4)		11.6 (4.2)		54.2 (11.0)	
Significantly below average	10 (4.0%)	25.4 (7.9)	**<0.001**	15.3 (10.6)	**<0.001**	73.5 (19.7)	**<0.001**	11.7 (9.1)	**<0.001**	10.4 (4.8)	**0.007**	54.0 (8.4)	**<0.001**
F			6.103		6.479		6.746		8.040		4.214		4.140
ω^2^			0.084		0.090		0.102		0.122		0.069		0.060
**Employment status**													
Fully employed	87 (34.8%)	17.5 (6.9)		24.9 (7.3)		93.1 (13.2)		22.7 (6.5)		7.1 (3.8)		43.8 (12.9)	
Partially employed	54 (21.6%)	20.3 (5.7)		20.7 (7.0)		85.5 (13.1)		19.4 (7.1)		9.7 (4.3)		52.1 (8.4)	
Student	15 (6.0%)	21.8 (5.0)		21.6 (7.3)		88.9 (14.3)		22.1 (5.9)		9.6 (4.1)		53.9 (6.5)	
Homemaker	2 (0.8%)	22.0 (-)		28.0 (-)		84.0 (-)		-		-		-	
Unemployed	7 (2.8%)	23.5 (5.1)		16.7 (7.5)		72.8 (14.8)		14.4 (8.7)		9.2 (5.4)		55.0 (7.0)	
On a leave of absence due to illness	5 (2.0%)	21.4 (5.5)		21.4 (8.8)		88.8 (17.8)		17.0 (7.2)		10.6 (4.0)		59.0 (4.2)	
Fully disabled and unemployed	59 (23.6%)	21.3 (7.0)		19.9 (9.1)		85.2 (14.2)		18.6 7.7)		10.3 (3.5)		52.4 (10.4)	
Retired	21 (8.4%)	18.5 (3.7)	**0.013**	22.6 (8.0)	**0.005**	90.5 (8.2)	**0.005**	25.3 (6.0)	**0.001**	9.3 (2.5)	**<0.001**	42.7 (9.4)	**<0.001**
F			2.618		2.973		3.012		4.196		3.819		6.408
ω^2^			0.048		0.059		0.065		0.087		0.089		0.142
**Education level**													
Elementary	1 (0.4%)	23.0 (-)		15 (-)		87.0 (-)		24.0 (-)		13.0 (-)		50.0 (-)	
Secondary	51 (20.4%)	20.4 (5.7)		20.5 (7.9)		82.7 (13.2)		19.4 (8.3)		8.9 (4.0)		51.7 (8.9)	
Above secondary	62 (24.8%)	20.2 (6.5)		20.4 (7.8)		85.5 (14.1)		18.6 (7.0)		9.1 (3.8)		49.7 (12.1)	
Academic	136 (54.4%)	18.9 (6.5)	0.437	23.6 (7.5)	**0.022**	91.5 (14.1)	**0.002**	22.2 (6.8)	**0.021**	8.8 (4.2)	0.746	47.6 (11.8)	0.260
F			0.909		3.272		5.102		3.511		0.377		1.282
ω^2^			−0.001		0.030		0.057		0.036		−0.011		0.004
**Total Mean**		**19.6 (6.4)**		**22.1 (7.8)**		**88.3 (13.8)**		**20.8 (7.3)**		**8.9 (4.0)**		**48.9 (11.5)**	

^A^ Of 270 respondents, 259 met all four inclusion criteria: age above 18, residence in Israel, the ability to answer independently in Hebrew, and a confirmed diagnosis of multiple sclerosis. Not all participants completed the whole questionnaire, but each separate scale received at least 201 valid responses. ^B^ Stress was quantified through the 10-item Perceived Stress Scale (PSS-10), Resilience through the Connor-Davidson 10-item Scale (CD-RISC-10), Well-being through the 18-Item Ryff’s Psychological Well-being, Satisfaction with Life through the 5-item Satisfaction with Life Scale, Sleep Quality through the Pittsburgh Sleep Quality Index, and Fatigue through the Fatigue Severity Scale (9-item). The significant *p*-values are presented in bold.

**Table 2:** Mobility and Daily Functioning in relation to the dependent variables.

Mobility and Daily Functioning	N (%)	Stress		Resilience		Well-Being		Satisfaction with Life		Sleep Quality		Fatigue	
None or minimal symptoms ^A^	17 (7%)	15.0 (7.6)		24.2 (7.0)		93.4 (14.5)		22.9 (6.3)		5.6 (3.7)		37.0 (14.4)	
Noticeable symptoms but no limitations ^B^	99 (40.7%)	19.3 (5.8)		23.2 (7.5)		90.3 (13.5)		21.7 (6.7)		7.7 (3.7)		47.6 (11.1)	
Moderate symptoms that affect daily functioning ^C^	65 (26.7%)	20.2 (6.3)		20.8 (6.8)		85.8 (13.6)		19.9 (7.9)		9.7 (3.6)		52.3 (9.2)	
Significant symptoms and support needed for walking ^D^	31 (12.8%)	22.1 (7.0)		19.0 (9.9)		83.7 (16.3)		17.3 (8.0)		10.4 (3.3)		53.2 (11.3)	
Significant symptoms and using a walker ^E^	24 (9.9%)	18.9 (6.5)		22.8 (7.9)		89.8 (12.5)		23.1 (5.9)		10.6 (5.2)		48.3 (9.0)	
Severe symptoms and using a wheelchair ^F^	7 (2.9%)	20.4 (5.1)		24.7 (7.7)		90.5 (7.7)		19.6 (7.6)		13.2 (2.5)		39.0 (16.5)	
*p*-value			**0.02**		0.082		0.117		**0.04**		**<0.001**		**<0.001**
F			2.745		1.982		1.788		2.384		5.954		5.697
ω^2^			0.038		0.022		0.019		0.033		0.125		0.107

^A^ I have no or minimal multiple sclerosis-related symptoms, no limitations in walking ability and no limitations on daily activities. ^B^ I have noticeable multiple sclerosis–related symptoms but no limitations in walking ability and no limitations on daily activities. ^C^ I have many multiple sclerosis–related symptoms that affect my daily activities but can walk at least 1 block without support. ^D^ I have significant multiple sclerosis–related symptoms that limit physically demanding activities. I need support (e.g., cane, touching a wall, leaning on someone’s arm) to walk ½ to 1 block. ^E^ I have significant multiple sclerosis–related symptoms that limit daily activities. I can walk only short distances with a walker or 2-handed crutches. ^F^ I have many severe multiple sclerosis–related symptoms and am restricted to a wheelchair or bed. The significant *p*-values are presented in bold.

**Table 3:** Disease-related characteristics in relation to the dependent variables.

Multiple Sclerosis Characteristics	N (%) ^A^	Stress ^B^ Mean (SD) *p*-Value t/F d or ω^2^		Resilience ^B^ Mean (SD) *p*-Value t/F d or ω^2^		Well-Being ^B^ Mean (SD) *p*-Value t/F d or ω^2^		Satisfaction with Life ^B^ Mean (SD) *p*-Value t/F d or ω^2^		Sleep Quality ^B^ Mean (SD) *p*-Value t/F d or ω^2^		Fatigue ^B^ Mean (SD) *p*-Value t/F d or ω^2^	
**Time since MS diagnosis**													
Less than two years	55 (22.6%)	21.4 (7.4)		21.4 (8.4)		87.7 (15.4)		20.0 (7.8)		8.7 (4.2)		48.9 (10.5)	
Between 2 to 5 years	56 (23.0%)	20.6 (5.2)		21.5 (7.0)		86.5 (12.0)		19.0 (7.2)		8.5 (4.0)		51.1 (10.7)	
Between 5 to 10 years	51 (21.0%)	18.4 (6.5)		21.3 (7.6)		88.3 (14.9)		21.9 (7.5)		9.4 (3.9)		49.9 (10.3)	
Between 10 to 20 years	47 (19.3%)	18.2 (6.2)		23.1 (7.9)		88.7 (14.2)		21.1 (6.6)		8.9 (4.0)		48.8 (12.3)	
More than 20 years	34 (14.0%)	18.2 (6.2)	**0.039**	23.6 (7.9)	0.55	91.3 (12.0)	0.703	22.4 (6.6)	0.285	9.3 (4.4)	0.654	44.4 (13.6)	0.208
F			2.573		0.764		0.545		1.457		0.335		1.624
ω^2^			0.028		−0.004		−0.009		0.009		−0.016		0.013
**Multiple Sclerosis Type**													
Relapsing Remitting (RRMS)	154 (63.1%)	19.1 (6.6)		22.2 (7.5)		88.8 (13.9)		20.7 (6.9)		8.7 (4.1)		48.5 (12.0)	
Secondary Progressive (SPMS)	23 (9.4%)	18.4 (5.3)		24.0 (7.7)		91.9 (14.6)		21.9 (7.7)		9.6 (4.2)		46.4 (12.2)	
Primary Progressive (PPMS)	25 (10.2%)	21 (7.3)		21.2 (8.2)		86.7 (12.8)		21.0 (9.6)		10.1 (4.0)		50.6 (9.4)	
Clinically Isolated Syndrome (CIS-MS)	14 (5.7%)	20.8 (5.5)		25.1 (8.1)		88.8 (13.3)		21.8 (6.3)		8.7 (4.3)		48.5 (7.3)	
Unknown to the participant	28 (11.5%)	21.7 (5.0)	0.237	18.5 (8.1)	0.081	83.3 (13.3)	0.320	18.9 (7.6)	0.715	8.7 (3.7)	0.450	52.4 (10.4)	0.412
F			1.395		2.107		1.181		0.483		0.783		0.836
ω^2^			0.007		0.020		0.004		−0.010		−0.005		−0.003
**Time since the last exacerbation**													
Less than 3 months	30 (12.8%)	20.5 (5.8)		22.1 (8.5)		88.1 (15.1)		20.7 (7.9)		11.8 (3.7)		53.3 (9.9)	
Between 3 months and a year	59 (25.2%)	21.8 (6.1)		20.9 (8.1)		85.4 (13.5)		19.2 (7.5)		8.8 (3.6)		52.0 (9.5)	
Between 1 to 3 years	79 (33.8%)	18.8 (6.9)		22.1 (6.7)		88.6 (13.5)		21.2 (7.2)		8.1 (3.7)		47.5 (10.6)	
More than 3 years	66 (28.2%)	18.4 (6.9)	**0.016**	22.6 (8.5)	0.695	90.2 (13.9)	0.381	21.1 (7.2)	0.501	8.5 (4.3)	**0.002**	46.8 (13.7)	**0.015**
F			3.517		0.482		1.029		0.789		5.176		3.298
ω^2^			0.034		−0.007		0.00		−0.003		0.069		0.035
**Comorbidities ^C^**													
At least 1	135 (54%)	20.7 (6.2)		21.0 (8.3)		86.7 (14.6)		19.9 (7.5)		9.4 (4.0)		50.7 (10.0)	
None	115 (46%)	18.2 (6.4)	**0.003**	23.5 (6.9)	**0.01**	90.5 (14.5)	**0.024**	22.0 (6.8)	**0.018**	8.3 (4.0)	0.091	46.5 (12.8)	**0.005**
t			−2.989		2.343		1.991		2.119		−1.701		−2.579
Cohen’s d			−0.402		0.318		0.283		0.302		−0.260		−0.371
**Mobility and Functioning (self-rated) ^D^**													
No disability, no or minor symptoms	116 (47.7%)	18.7 (6.2)		23.3 (7.4)		90.7 (13.6)		21.9 (6.6)		7.4 (3.8)		46.2 (12.0)	
Any level of disability	127 (52.3%)	20.4 (6.5)	**0.02**	21.0 (7.9)	**0.012**	86.1 (13.8)	**0.01**	19.8 (7.8)	**0.021**	10.2 (3.8)	**<0.001**	51.3 (10.4)	**<0.001**
t			−2.064		2.277		2.353		2.048		−4.797		−3.141
Cohen’s d			−0.277		0.307		0.332		0.288		−0.731		−0.449
**Diagnosed Mental Health Conditions ^E^**													
Anxiety	40 (16.0%)	23.2 (6.0)	**<0.001**	16.6 (7.5)	**<0.001**	81.2 (13.6)	**<0.001**	16.3 (8.4)	**<0.001**	10.3 (3.1)	**0.034**	54.7 (7.2)	**<0.001**
t			−3.767		4.904		3.316		3.907		−1.836		−3.166
Cohen’s d			0.693		0.893		0.631		0.753		−0.411		−0.620
Depression	41 (16.4%)	24.5 (6.5)	**<0.001**	15.6 (7.6)	**<0.001**	77.9 (13.1)	**<0.001**	15.2 (6.9)	**<0.001**	10.8 (3.8)	**0.007**	56.5 (6.1)	**<0.001**
t			−5.405		6.067		5.031		5.050		−2.504		−4.202
Cohen’s d			−0.973		1.0		0.957		0.961		−0.541		−0.823
**On Disease Modifying Therapy**													
Yes	203 (83.2%)	20.0 (6.2)		21.8 (7.4)		88.2 (14.2)		20.7 (7.3)		9.0 (4.2)		49.0 (11.4)	
No	41 (16.8%)	19.5 (6.5)	0.652	23.6 (9.2)	0.09	89.0 (12.0)	0.754	21.0 (7.3)	0.822	8.7 (3.3)	0.757	48.0 (11.8)	0.602
t			0.452		1.329		0.314		0.225		−0.310		−0.461
Cohen’s d			0.081		0.240		0.058		0.042		−0.066		−0.089
**Experiencing MS symptoms last week ^F^**													
Yes	210 (86.1%)	20.0 (6.3)		21.9 (7.9)		87.7 (13.6)		20.3 (7.3)		9.4 (4.0)		50.4 (10.2)	
No	34 (13.9%)	16.5 (6.6)	**0.006**	22.9 (6.9)	0.29	92.8 (14.7)	**0.041**	24.2 (5.9)	**0.005**	6.2 (3.1)	**<0.001**	38.5 (14.5)	**<0.001**
t			−2.773		0.553		1.752		2.571		−3.734		−5.148
Cohen’s d			−0.560		0.114		0.374		0.540		−0.821		−1.102
**Have seen a therapist post-diagnosis**													
Yes	112 (46.3%)	20.8 (6.1)		20.7 (7.2)		86.1 (14.5)		20.0 (7.2)		9.5 (3.9)		52.1 (9.5)	
No	130 (53.7%)	18.6 (6.4)	**0.005**	23.3 (8.1)	**0.006**	90.2 (13.1)	**0.021**	21.4 (7.3)	0.081	8.5 (4.1)	0.075	46.4 (12.3)	**<0.001**
t			−2.571		2.530		2.049		1.400		−1.612		−3.551
Cohen’s d			−0.347		0.343		0.290		0.198		−0.246		−0.511
**Have participated in MS group support**													
Yes	54 (77.9%)	20.1 (6.4)		20.4 (8.5)		85.3 (14.0)		19.6 (7.0)		10.0 (3.7)		48.9 (12.4)	
No	190 (22.1%)	19.5 (6.4)	0.523	22.5 (7.5)	**0.05**	89.1 (13.7)	0.057	21.1 (8.3)	0.123	8.6 (4.1)	**0.029**	48.8 (11.2)	0.797
t			−0.639		1.651		1.584		1.162		−1.902		−0.028
Cohen’s d			−0.105		0.271		0.277		0.200		−0.343		−0.005

^A^ Of 270 respondents, 259 met all four inclusion criteria: age above 18, residence in Israel, the ability to answer independently in Hebrew, and a confirmed diagnosis of multiple sclerosis. Not all participants completed the whole questionnaire, but all separate scales received at least 196 valid responses. ^B^ Stress was quantified through the 10-item Perceived Stress Scale (PSS-10), Resilience through the Connor-Davidson 10-item Scale (CD-RISC-10), Well-being through the 18-Item Ryff’s Psychological Well-being, Satisfaction with Life through the 5-item Satisfaction with Life Scale, Sleep Quality through the Pittsburgh Sleep Quality Index, and Fatigue through the Fatigue Severity Scale (9-item). ^C^ The diagnoses included: hypercholesterolemia (32), autoimmune disorders (30), thyroid disorders (20), hypertension (16), diabetes (12), cancer (5), and fibromyalgia (4). Other diagnoses included: cardiovascular disease (3), psoriasis (3), epilepsy (3), COPD (1), tachycardia (1), osteoporosis (1), chronic migraines (1), anemia (1), Darier’s Disease (1), arthritis (1), and irritable bladder (1). ^D^ The perceived ability and disability levels were rated by the participants using the following descriptions (as translated from Hebrew): TABLE X. ^E^ In addition to depression and anxiety, 3 persons reported being diagnosed with attention deficit disorder and 2 people with borderline personality disorder. ^F^ The following list of symptoms was included, as examples of the most common MS symptoms: double vision; unintentional movement of the eyes; inability to focus one’s sight; temporary loss of sensation; heaviness or weakness unrelated to physical exertion; a sensation of tingling or “electric currents”; tics; extreme fatigue or inability to focus; nerve pain; migraines; unrelenting itching; an unusual sensation of hot or cold; dizziness; loss of coordination or balance; falls; bladder control issues; and digestive issues unrelated to a food allergy or sensitivity. The significant *p*-values are presented in bold.

**Table 4:** Correlations between the dependent variables of the study as well as age.

Variables	Stress	Resilience	Well-Being	Satisfaction with Life	Sleep Quality	Fatigue	Age
Stress	-						
Resilience	−0.641 **	-					
Well-being	−0.627 **	0.699 **	-				
SwL	−0.559 **	0.573 **	0.775 **	-			
Sleep Quality	0.308 **	−176 *	−0.166	−0.177 *	-		
Fatigue	0.543 **	−0.463 **	−0.418 **	−0.403 **	0.326 **	-	
Age	−0.212 **	0.138 *	0.126	0.142 *	0.110	−0.154 *	-

Significant correlation: * *p* < 0.05; ** *p* < 0.001.

**Table 5:** Perceived Stress: a multivariable linear model.

Factor	Unstandardized Coefficients		Standardized Coefficients	t	Sig.
	B	Std. Error	Beta		
Gender	−0.462	0.813	−0.027	−0.856	0.393
Age	−0.016	0.027	−0.029	−0.569	0.570
PWB: Environmental Mastery	−0.467	0.129	−0.275	−3.627	<0.001
PWB: Self-acceptance	−0.239	0.107	−0.141	−2.239	0.027
Resilience	−0.313	0.062	−0.350	−5.023	<0.001
Sleep Quality	0.211	0.084	0.127	2.499	0.013
Fatigue	0.075	0.033	0.130	2.290	0.023
Clinically Isolated Syndrome	3.620	1.425	0.120	2.541	0.012

**Table 6:** Resilience: a multivariable linear model.

Factor	Unstandardized Coefficients		Standardized Coefficients	t	Sig.
	B	Std. Error	Beta		
Gender	−1.156	0.876	−0.060	−1.320	0.188
Age	0.003	0.031	0.005	0.188	0.692
Perceived Stress	−0.398	0.077	−0.330	−5.171	<0.001
Anxiety	−3.462	0.999	−0.164	−3.645	<0.001
PWB: Autonomy	0.272	0.116	0.110	2.341	0.020
PWB: Environmental Mastery	0.486	0.136	0.241	3.565	<0.001
PWB: Personal Growth	0.824	0.146	0.290	5.650	<0.001

**Table 7:** Sleep Quality: a multivariable linear model.

Factor	Unstandardized Coefficients		Standardized Coefficients	t	Sig.
	B	Std. Error	Beta		
Gender	−0.223	0.697	−0.022	−0.319	0.750
Age	0.040	0.024	0.1114	1.634	0.104
Perceived Stress	0.166	0.042	−0.277	3.923	<0.001
MS Symptoms last week	2.283	0.821	0.191	2.780	0.006
Less than 3 months since exacerbation	2.501	0.806	0.213	3.104	0.002
No disability, some symptoms	−1.434	0.556	−0.174	−2.581	0.011
Below = average income	1.867	0.858	0.150	2.177	0.031
PWB: Autonomy	0.216	0.088	0.167	2.457	0.015

## Data Availability

The data presented in this study are available on request from the corresponding author.

## Supplementary Materials

The following supporting information can be downloaded at: https://www.mdpi.com/article/10.3390/jcm12020716/s1.
