# Supplementary material for: Resilience, Stress, Well-Being, and Sleep Quality in Multiple Sclerosis
Source: J Clin Med. 2023 Jan 16;12(2):716. doi: 10.3390/jcm12020716 (PMC9864697; doi:10.3390/jcm12020716)
Supplement: Supplementary file 1 [file jcm-12-00716-s001.zip › jcm-2103600-supplementary.pdf]

Resilience, stress, well-being, and sleep quality in Multiple Sclerosis
Supplementary File

Contents:

1. Perceived Stress Linear Regression Model
2. Resilience Linear Regression Model
3. Sleep Quality Linear Regression Model
4. Alpha Cronbach Values for all of the questionnaires used in the study
5. The baseline characteristics questionnaire of the study, excluding the separate, validated scales.

1. Perceived Stress Linear Regression Model

Model Summary[b]

Model	R	R Square	Adjusted R Square	Std. Error of the Estimate	Change Statistics				
					R Square Change	F Change	df1	df2	Sig. F Change
1	.809[a]	.655	.638	4.09348	.655	37.770	8	159	<.001

a. Predictors: (Constant), A12=MS-CIS אבחנה על סמך התקף קליני בודד עם עדות לטרשת נפוצה בבדיקת MRI, PSQI_total, מה המגדר שלך?, age, R_S_Acceptance, Fatigue, Resilience, R_E_Mastery

b. Dependent Variable: PSS_total

ANOVA[a]

Model		Sum of Squares	df	Mean Square	F	Sig.
1	Regression	5063.220	8	632.903	37.770	<.001[b]
	Residual	2664.298	159	16.757		
	Total	7727.518	167			

a. Dependent Variable: PSS_total

b. Predictors: (Constant), A12=MS-CIS אבחנה על סמך התקף קליני בודד עם עדות לטרשת נפוצה בבדיקת MRI, PSQI_total, מה המגדר שלך?, age, R_S_Acceptance, Fatigue, Resilience, R_E_Mastery

Coefficients

Model		Unstandardized Coefficients		Standardized Coefficients	t	Sig.
		B	Std. Error	Beta		
1	(Constant)	31.641	2.756		11.480	<.001
	Age	-.016	.027	-.029	-.590	.556
	Gender	-.462	.813	-.027	-.569	.570
	Environmental Mastery	-.467	.129	-.275	-3.627	<.001
	Self-acceptance	-.239	.107	-.141	-2.239	.027
	Resilience	-.313	.062	-.350	-5.023	<.001
	Sleep Quality	.211	.084	.127	2.499	.013
	Fatigue	.075	.033	.130	2.290	.023
	MS-CIS	3.620	1.425	.120	2.541	.012

a. Dependent Variable: PSS_total

2. Resilience Linear Regression Model

Model Summary[b]

Model	R	R Square	Adjusted R Square	Std. Error of the Estimate	Change Statistics				
					R Square Change	F Change	df1	df2	Sig. F Change
1	.779[a]	.607	.593	4.94808	.607	42.423	7	192	<.001

a. Predictors: (Constant), R_P_Growth, anxiety, מה המגדר שלך?, R_Autonomy, age, PSS_total, R_E_Mastery

b. Dependent Variable: Resilience

ANOVA[a]

Model		Sum of Squares	df	Mean Square	F	Sig.
1	Regression	7270.584	7	1038.655	42.423	<.001[b]
	Residual	4700.836	192	24.484		
	Total	11971.420	199			

a. Dependent Variable: Resilience

b. Predictors: (Constant), R_P_Growth, anxiety, מה המגדר שלך?, R_Autonomy, age, PSS_total, R_E_Mastery

Coefficients

Model		Unstandardized Coefficients		Standardized Coefficients	t	Sig.
		B	Std. Error	Beta		
1	(Constant)	7.310	4.063		1.799	.074
	age	.003	.031	.005	.108	.914
	Gender	-1.156	.876	-.060	-1.320	.188
	Environmental Mastery	.486	.136	.241	3.565	<.001
	Perceived Stress	-.398	.077	-.330	-5.171	<.001
	Anxiety	-3.462	.999	-.164	-3.465	<.001
	Autonomy	.272	.116	.110	2.341	.020
	Personal Growth	.824	.146	.290	5.650	<.001

a. Dependent Variable: Resilience

3. Sleep Quality Linear Regression Model

Model Summary

Model	R	R Square	Adjusted R Square	Std. Error of the Estimate	Change Statistics				
					R Square Change	F Change	df1	df2	Sig. F Change
1	.573[a]	.329	.295	3.41847	.329	9.618	8	157	<.001

a. Predictors: (Constant), A8=מתחת לממוצע, האם חווית בשבוע האחרון תסמיני טרשת נפוצה?* *דוגמאות של תסמינים: בעיות ראיה
כולל איבוד ראייה, טשטוש, ראייה כפולה או תנועות בלתי נשלטות של העין, נימול בחלק גוף מסוים, כבדות או חולשה בשרירים לא מקושרת
למאמץ אינטנסיבי, עוויתות או טיקים, עייפות או חוסר יכו, A15=יש לי מספר תסמיני טרשת נפוצה אבל הם לא מגבילים אותי בהליכה
ובתפקוד היום-יומי שלי, R_Autonomy, מה המגדר שלך?, A13=פחות משלושה חודשים, age, PSS_total

ANOVA[a]

Model		Sum of Squares	df	Mean Square	F	Sig.
1	Regression	899.122	8	112.390	9.618	<.001[b]
	Residual	1834.691	157	11.686		
	Total	2733.813	165			

a. Dependent Variable: PSQI_total

b. Predictors: (Constant), A8=מתחת לממוצע, האם חווית בשבוע האחרון תסמיני טרשת
נפוצה?* *דוגמאות של תסמינים: בעיות ראיה, כולל איבוד ראייה, טשטוש, ראייה
כפולה או תנועות בלתי נשלטות של העין, נימול בחלק גוף מסוים, כבדות או חולשה
בשרירים לא מקושרת למאמץ אינטנסיבי, עוויתות או טיקים, עייפות או חוסר יכו,
A15=יש לי מספר תסמיני טרשת נפוצה אבל הם לא מגבילים אותי בהליכה ובתפקוד
היום-יומי שלי, R_Autonomy, מה המגדר שלך?, A13=פחות משלושה חודשים, age,
PSS_total

Coefficients

Model		Unstandardized Coefficients		Standardized Coefficients	t	Sig.
		B	Std. Error	Beta		
1	(Constant)	-1.118	2.099		-.532	.595
	age	.040	.024	.114	1.634	.104
	Gender	-.223	.697	-.022	-.319	.750
	Perceived Stress	.166	.042	.277	3.923	<.001
	Autonomy	.216	.088	.167	2.457	.015
	MS symptoms last week	2.283	.821	.191	2.780	.006
	Less than 3 months since exacerbation	2.501	.806	.213	3.104	.002
	No disability, some symptoms	-1.434	.556	-.174	-2.581	.011
	Below average income	1.867	.858	.150	2.177	.031

a. Dependent Variable: PSQI_total

4. Alpha Cronbach values

	Cronbach's Alpha	*Cronbach's Alpha based on standardized items*	*Number of items*
Perceived Stress Scale	**.855**	.886	10
Connor Davidson Resilience Scale	**.893**	.895	10
Satisfaction with Life Scale	**.884**	.884	5
Fatigue Severity Scale	**.924**	.927	9
Ryff's Psychological Well-being Scale	**.823**	.820	18
Pittsburgh Sleep Quality Index	**.659**	.664	14

5. The baseline characteristics questionnaire of the study, excluding the separate, validated scales.

TRANSLATED from the Hebrew

PART 1: Inclusion Criteria Assessment

Has a neurologist officially diagnosed you with Multiple Sclerosis?
- o Yes
- o No

Do you speak and read Hebrew and are you capable of answering independently?
- o Yes
- o No

Do you live and are treated for Multiple Sclerosis in Israel?
- o Yes
- o No

Are you over the age of 18?
- o Yes
- o No

PART 2: Sociodemographic factors

1. Age: ____________________

2. Gender:
Male / Female / Other

3. Marital status:
Single / In a common-law partnership / Married / Divorced / Widowed

4. Your financial situation as compared to other families:
Significantly below average / Below average / Average / Above average / Significantly above average

5. Employment status
Full-time (including self-employment) / Part-time (including self-employment) / Homemaker / Student / Unemployed / On a prolonged leave of absence due to illness / Fully disabled and unemployed / Pensioner

6. Education
Elementary / Secondary school / Above secondary / Academic

PART 3: Clinical factors

7. How much time has passed since your Multiple Sclerosis diagnosis?

- o Less than two years
- o Two to five years
- o Five to ten years
- o Ten to twenty years
- o More than twenty years

8. With which type of Multiple Sclerosis are you currently diagnosed?
- o Relapsing Remitting Multiple Sclerosis (RRMS)
- o Secondary Progressive Multiple Sclerosis (SPMS)
- o Primary Progressive Multiple Sclerosis (PPMS)
- o Clinically Isolated Syndrome Multiple Sclerosis (CIS-MS)
- o I do not know

9. How much time has passed since your most recent exacerbation* of Multiple Sclerosis?
- o Less than three months
- o Three months to one year
- o One to three years
- o More than three years

* A Multiple Sclerosis exacerbation is defined as an occurrence of a new symptom(s) or a significant worsening of a previously existing symptom of MS, that lasts at least 48 hours. During an exacerbation, the symptom(s) usually worsens over a period of days to weeks, and eventually resolves. An MS exacerbation is often treated using a course of steroids administered in a hospital setting. Some examples of MS exacerbations include vision loss, double vision, numbness, or loss of sensation in a limb, vertigo, problems with balance and coordination.

10. Do you receive Disease Modifying Therapy (a medication) for Multiple Sclerosis?
- o Yes
- o No

11. How would you describe your daily functioning?
- o I have no or minimal multiple sclerosis-related symptoms, no limitations in walking ability and no limitations on daily activities.
- o I have noticeable multiple sclerosis–related symptoms but no limitations in walking ability and no limitations on daily activities.
- o I have many multiple sclerosis–related symptoms that affect my daily activities but can walk at least 1 block without support.
- o I have significant multiple sclerosis–related symptoms that limit physically demanding activities. I need support (e.g., cane, touching a wall, leaning on someone's arm) to walk ½ to 1 block.
- o I have significant multiple sclerosis–related symptoms that limit daily activities. I can walk only short distances with a walker or 2-handed crutches.
- o I have many severe multiple sclerosis–related symptoms and am restricted to a wheelchair or bed

12. Have you experienced any Multiple Sclerosis symptoms this week*?
- o Yes
- o No

* Some examples of common Multiple Sclerosis symptoms include vision problems such as blurry vision, uncontrolled eye movements, double vision or loss of vision, paresthesia (pins and needles), muscle spasms and tics, loss of sensation, heaviness or muscle weakness that can't be explained by physical exertion, fatigue, inability to focus, nerve pain, migraine, unrelenting itchiness or tingling, dysesthesia (or an "electric shock" sensation), dizziness, loss of coordination or balance, falls, movement disorders, bladder disorders, digestive issues

13. Have you been diagnosed with any of these disorders? (Choose all that apply)

- Diabetes
- Hypertension
- Hypercholesterolemia
- Heart disease
- Thyroid disorders
- A different autoimmune disease
- Epilepsy
- Cancer
- Depression
- Anxiety
- Other ___________________________

14. Did you receive psychological counselling following the Multiple Sclerosis diagnosis?
- Yes
- No

15. Did the neurologist who is treating you for Multiple Sclerosis recommend you seek psychological counselling or refer you to a therapist or a social worker following the diagnosis of Multiple Sclerosis?
- Yes
- No

16. Do you participate or did you participate in the past year in support group sessions for People with Multiple Sclerosis, including virtual meetings?
- Yes
- No

ORIGINAL (in Hebrew)

<u>חלק 1. בדיקת קריטריונים להכללה.</u>

האם את.ה חולה טרשת נפוצה, עם אבחנה מאושרת ע"י נוירולוג בכיר מומחה טרשת נפוצה?
- כן
- לא

האם את.ה מדבר.ת וקורא.ת עברית ומסוגל.ת לענות לבד?
- כן
- לא

האם את.ה מתגורר.ת ומטופל.ת כעת בישראל?
- כן
- לא

האם גילך מעל 18?
- כן
- לא

<u>חלק 2. נתונים דמוגרפים.</u>

2. **גיל**: ___________________________

3. **מגדר:**

זכר / נקבה / אחר

4. **מצב משפחתי**:

רווק.ה / ידועים בציבור / נשוי.אה / גרוש.ה / אלמן.ה

5. **מצבך הכלכלי ביחס למשפחות אחרות**:

הרבה מתחת לממוצע/ מתחת לממוצע/ ממוצע/ מעל הממוצע / הרבה מעל הממוצע

6. **תעסוקה**:

משרה מלאה (גם עבודה כעצמאי) / משרה חלקית (גם עבודה כעצמאי) / עקר.ת בית

/ סטודנט.ית / מובטל.ת / בחופשה ממושכת בעקבות מחלה / לא עובד כלל בעקבות מחלה או נכות / גמלאי

7. **השכלה**:

יסודית / תיכונית / על-תיכונית / אקדמאית

<u>חלק 3. נתונים בסיסיים על המחלה.</u>

8. כמה זמן עבר מאז אבחנתך כחולה טרשת נפוצה?
   - פחות משנתיים
   - בין 2-5 שנים

- בין 5-10 שנים
- בין 10 ל-20 שנה
- מעל 20 שנה

9. עם איזה סוג טרשת נפוצה את.ה מאובחן.ת כעת?
- טרשת נפוצה התקפית-הפוגתית RRMS
- טרשת נפוצה מתקדמת משנית SPMS
- טרשת נפוצה מתקדמת ראשונית PPMS
- אבחנה על סמך התקף קליני בודד עם עדות לטרשת נפוצה ב-MRI, MS-CIS
- אני לא יודע.ת

10. כמה זמן עבר מאז התקף* טרשת נפוצה האחרון שלך?
- פחות משלושה חודשים
- בין שלושה חודשים לשנה
- בין שנה לשלוש שנים
- יותר משלוש שנים

[*הסבר: התקף טרשת נפוצה הינו התפתחות של תסמין חדש או החמרה קשה של תסמין ישן, שנמשכת יותר מ-48 שעות. במהלך התקף, התסמין לרוב מחמיר במשך ימים עד שבועות ולאחר מכן משתפר באופן הדרגתי. לרוב, התקף דורש טיפול תרופתי בבית חולים בעזרת סטרואידים. דוגמא להתקף טרשת נפוצה: איבוד ראיה בעין אחת, איבוד תחושה ברגל או ביד, איבוד שיווי משקל.]

11. האם את.ה מקבל.ת טיפול להאטת התקדמות טרשת נפוצה (תרופה)?
- כן
- לא

12. איך היית מתאר.ת את רמת התפקוד שלך?
- אין לי בכלל תסמיני טרשת נפוצה ואינני מוגבל.ת בכלל בהליכה ובתפקוד היום-יומי שלי
- יש לי מספר תסמיני טרשת נפוצה אבל הם לא מגבילים אותי בהליכה ובתפקוד היום-יומי שלי
- יש לי הרבה תסמיני טרשת נפוצה שמשפיעים על התפקוד היום-יומי שלי, אבל אני יכול.ה ללכת לפחות למרחק של 200 מטר באופן עצמאי לחלוטין.
- אני זקוק.ה לעזרה קלה בהליכה ואני סובלת מתסמינים משמעותיים של טרשת נפוצה שמגבילים אותי ביום-יום. אני משתמש.ת בעזרים (כגון מקל, הליכה ליד קיר) או מבקש.ת עזרה מאחרים בשביל להתנייד.
- אני משתמש.ת בהליכון ויכול.ה ללכת בעזרתו רק למרחק קצר. ויש לי תסמינים משמעותיים שמגבילים אותי באופן מאוד ביום-יום.
- אני לא מסוגל.ת ללכת כלל ואני בכיסא גלגלים או במיטה. יש לי תסמינים רבים וקשים של טרשת נפוצה שמגבילים אותי באופן משמעותי מאוד.

13. האם חווית בשבוע האחרון תסמיני טרשת נפוצה*?
- כן
- לא

[*דוגמאות של תסמינים: בעיות ראיה, כולל איבוד ראייה, טשטוש, ראייה כפולה או תנועות בלתי נשלטות של העין, נימול בחלק גוף מסוים, כבדות או חולשה בשרירים לא מקושרת למאמץ אינטנסיבי, עוויתות או טיקים, עייפות או חוסר יכולת להתרכז, כאב, כולל כאבי ראש ומיגרנות, תחושת גרד, זרם חשמלי, עקצוצים, קור או חום, סחרחורת או רעד, חוסר קואורדינציה , חוסר שיווי משקל, הפרעות בתנועה או נפילות, הפרעות בשלפוחית השתן, הפרעות בעיכול כמו עצירות]

14. האם אובחנת עם אחת או יותר ממחלות הבאות? (נא לסמן את כל המחלות הרלוונטיות)
- סכרת
- יתר לחץ דם
- כולסטרול גבוה
- מחלות לב
- מחלות בלוטת התריס
- מחלה אוטואימונית אחרת
- אפילפסיה

- סרטן
- דיכאון
- חרדה
- אחר ______________

15. האם קיבלת יעוץ פסיכולוגי בעקבות האבחנה של טרשת נפוצה?
    - כן
    - לא

16. האם רופא נוירולוג שלך המליץ לך ו/או הפנה אותך לפסיכולוג.ית או עובד.ת סוציאלי.ת לאחר אבחנת טרשת נפוצה?
    - כן
    - לא

17. האם את.ה משתתפ.ת או השתתפת בשנה האחרונה במפגשי קבוצת תמיכה לחולי טרשת נפוצה, כולל מפגשים אינטרנטיים?
    - כן
    - לא
